# Xenotransplantion: Defeating the “Shumway Curse” An Interview With Drs. Bartley Griffith, Jayme Locke, Robert Montgomery, and Bruno Reichart

**DOI:** 10.3389/ti.2022.10439

**Published:** 2022-03-30

**Authors:** Thierry Berney, Maarten Naesens, Stefan Schneeberger

**Affiliations:** Transplant International

**Dr. Robert Montgomery, is a Professor and Chair of Surgery, and Director of the Transplant Institute at New York University (NYU) Langone Medical Center, United States.**

On Sep. 25, 2021, Dr. Montgomery transplanted a kidney from a genetically modified pig into a deceased human body donor, an experiment he successfully reproduced a few weeks later. Urine production could be observed for up to 3 days.

**Dr. Jayme Locke, is a Professor of Surgery and Director of the Comprehensive Transplant Institute and Division of Transplantation at the University of Alabama at Birmingham (UAB), United States.**

On Sep. 30, 2021, Dr. Locke transplanted two genetically modified pig kidneys inside the abdomen of a brain-dead human after removing the recipient's native kidneys. Urine production for over 72 hours was also observed (1).

**Dr. Bartley Griffith, is a Professor of Surgery and Director of the Cardiac and Lung Transplant Programs at the University of Maryland School of Medicine, Baltimore, United States.**

On Jan 7, 2022, Dr. Griffith performed the first successful xenogeneic heart transplant from a genetically modified pig to a human.

**Dr. Bruno Reichart, is an Emeritus Professor of Surgery and project leader at the Walter Brendel Center for Experimental Medicine, Ludwig-Maximilian University (LMU), Munich, Germany.**

Dr. Reichart performed Germany's first successful heart transplant in 1981, and has been a leading scientist and a spokesman for experimental xenotransplantation since over 20 years.

**It is commonplace to quote Dr. Norman Shumway as saying that “xenotransplantation is the future and always will be.” You and your teams seem to have successfully challenged this prediction. You have achieved and reported, in a super rapid sequence, what we feel is one of the most exciting breakthroughs in transplantation medicine since the turn of the century.**

**The key to these successes seems to have been the availability of pigs genetically engineered to evade xenogeneic rejection mechanisms (1)**.

BG: D. Craig Miller (note: cardio-thoracic surgery pioneer from Stanford) wrote to me after our news broke. He congratulated us. He went on to chuckle over this well-traveled quote of his mentor Norman Shumway. He felt that if Norm had lived to see cloning of mammals and CRISPR gene editing, he might have softened his negativity. Further, he told me that Norm would have appreciated the lengthy study period and substantial animal trials that told of the group’s preparedness. Today we are POD#24, so it is way early to conclude on long-term success, but the heart is doing quite well (note: the interview was conducted on 1 February 2022; since then, the death of the recipient, apparently for reasons unrelated to rejection, was communicated by the Maryland team).

**Figure:**
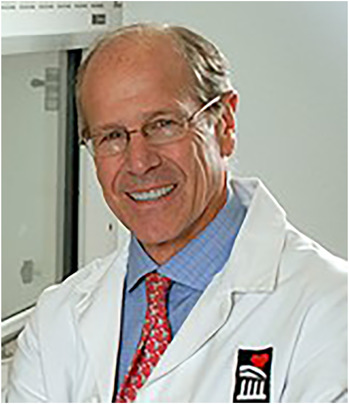
Dr. Bartley Griffith (BG).

**Q: Can you tell us what are the molecules/pathways that had to be targeted to produce organs that would have a reasonable chance of escaping the immunologic hurdles of xenotransplantation? How many genes had to be inserted/knocked-out? Have you used the same porcine “strains” and are the genes to target different for kidney or heart?**

JL, BG: At University of Alabama and University of Maryland, we transplanted kidneys from a pig donor with 10 gene edits (10 GE). Four genes were knocked out: three related to carbohydrate antigens known to cause hyperacute rejection (α 1-3 Gal, β 1-4 Gal, CMAH) and one involved the deletion of the pig growth hormone receptor (GHR). There were also six human transgene insertions. These edits were designed to further modulate the human immune system to help decrease inflammation (hCD47, hHO-1), and regulate complement (hCD46, hDAF) and coagulation (human thrombomodulin, human endothelial protein C receptor) (2).

BG: The goal of Revivicor/United Therapeutics (note: the company producing the genetically modified porcine donors for the Maryland transplant and the Birmingham experiment) is to have a single commercial pig for all organs if possible.

RM: We have taken the approach that “less is more.” α 1-3 Gal has always been the clear barrier to xenotransplantation with up to 1% of total human immunoglobulin targeting this epitope. We have crossmatched a large number of patients against α 1-3 Gal KO pigs and most have very reasonable crossmatches (note: α 1-3 Gal KO (GalSafe) pigs were recently developed and approved by the FDA, primarily as a source of allergy safe meat). A lot of the transgenes that are being dropped into the pig genome have variable expression and targets that can be regulated by drugs that are approved for human use.

I do think different organs are going to have different genetic engineering requirements. The challenge will be designing trials that can test individual “knock outs” and “knock ins” so we don’t just accumulate complex constructs with unproven individual components.

**Figure:**
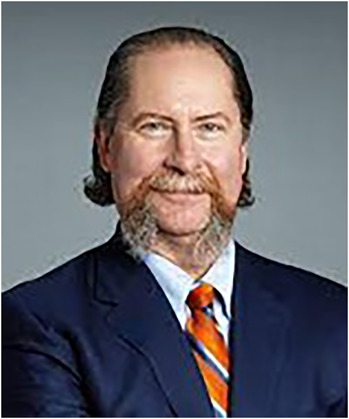
Dr Robert Montgomery (RM).

BR: For pig-to-baboon models, knocking out α 1-3 Gal is sufficient. This is enough to prevent hyperacute rejection, but when moving to the human, you need to target additional genes, as explained by Drs. Locke and Griffith. One major concern is that pigs grow to sizes much larger than humans, and the size of an organ in an animal is genetically determined by the size of the adult body. This made it necessary to knock-out the human GHR to prevent the donor organ from outgrowing its new human host. One problem with GHR-KO animals is that their reproduction is affected and they are therefore very difficult to breed. Because of the breeding difficulties, among other reasons, the UAB and Maryland groups have utilized cloned, as opposed to reproduced, animals.

I agree with Dr. Montgomery that it may be preferable to change as little as possible in the genes of the porcine donors and utilize pharmacological agents (e.g., inhibitors, monoclonals,…) rather than extensive genetic modifications. As for the size issue, instead of knocking-off GHR, the Munich group is looking for smaller breeds of pigs that only grow to 70–80 kg.

**Q: How long and how did you prepare yourselves and your teams before taking the final step? How much of a leap of faith has it been?**

BG: Dr. Muhammad Mohiuddin has been studying xenoheart for nearly 3 decades. He developed the immunosuppression protocol in pig-to-baboon in an intra-abdominal implant model. His 3-year success was a major factor in his relocation from the NIH to the University of Maryland 5 years ago. Our goal has been to translate his work to a model that would directly translate to humans. We focused on orthotopic xenoheart transplantation in 18–30 kg baboons. We refined our processes and approach. We have consistent survivals of 3 months and beyond with minimal evidence of rejection. Our longest animal was going strong at 9 months, when he died of respiratory failure from a presumed virus that hit other animals in the post-operative care facility.

Prior to making an incision in our patient the team took a quiet moment of reflection to give thanks and a hope for help in our quest. We were confident the time was right to try.

JL: We launched the University of Alabama Xenotransplant Program in 2016. We invested heavily in building out the necessary infrastructure in order to make xenotransplantation a reality for the many patients in need. This included the development and implementation of a pathogen-free facility in which the donor source animal (pig) can be bred and raised in an environment that decreases/eliminates the risk of viral or other disease transmission to human recipients from these pig organs. We also simultaneously continued to study the 10 GE pig kidney transplant in non-human primates (NHP). However, it became very clear that the NHP model was not immunologically similar enough to humans to answer key safety questions: would hyperacute rejection be avoided in the setting of standard immunosuppression used in allo-human-to-human transplantation? would viral transmission or chimerism develop in a human host? would the pig vasculature withstand adult human arterial pressure? and importantly the NHP model was insufficient to validate our novel flow crossmatch designed to predict tissue compatibility a priori (e.g., prior to transplant as is required by federal regulators for human-to-human transplantation). We therefore sought a novel pre-clinical human model—human brain death. The implementation of this model began at UAB 2 years ago. We initiated an external ethics review and engaged our Institutional Review Board (IRB). Although by definition the model is not human subject research and therefore not under the purview of an IRB, we felt given the magnitude of the study and our goals of recapitulating every step in the process that would be necessary to move this into living people, we elected to perform the study under IRB approval. As with all science, there is some element of a “leap of faith,” but in reality the UAB study was hypothesis-driven to answer early endpoints that simply could not be answered in a NHP model, and were absolutely critical prior to risking the life of a living person—e.g., demonstration that we could assess tissue compatibility a priori, demonstrate no hyperacute rejection in the setting of conventional immunosuppression, and ensure no porcine endogenous retroviruses (PERV) were transmitted.

**Figure:**
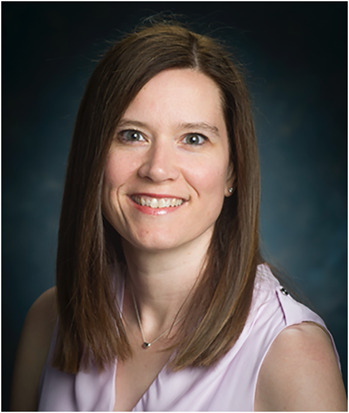
Dr Jayme Locke (JL).

RM. We have been working on setting up the studies in the recently deceased for 5 years. It required a lot of vetting and regulatory work. Honestly, when we did the first transplant on 25 September 2021, I said to the team just before we removed the vascular clamps, “I don’t know what is going to happen here but we will learn something very important today.”

**Q: These reports have almost taken the transplant community by surprise. Do you see these as a quantum leap or as the natural progression of xenotransplantation research over the past decades? How much of the success is due to the discovery CRISPR/Cas9 genome editing system of Nobel prize fame?**

BG: The coming together in cases is likely a response to the availability of edited pigs. Certainly, gene editing is a huge accelerant.

JL: We believe these advances represent the natural progression of xenotransplantation research, which has most definitely benefited from the introduction of CRISPR/Cas9 genome editing allowing for greater precision and rapidity.

RM: The α-Gal KO in the pig we used was created by homologous recombination. I think the big leap was made possible by the concept of whole-body donation for the purpose of high stakes studies like xenotransplantation as an intermediate step between animal and phase I trials. This could be done without the risk of harm to the patient since they were brain dead. I think once we did that the genie was out of the bottle.

**Q: Is transmission of porcine retroviruses (PERV) still a problem or has it ever been? How, if at all, have you tackled it?**

JL: PERVs have long been a concern in the field, but these concerns have evolved over time as well. Regulators now acknowledge that PERV A and B are endemic in pig herds and that it is PERV C that is known to cause disease in humans. The donor source pigs at UAB are housed in a pathogen free facility and undergo routine biosurveillance. They are negative for PERV C. Importantly, our deceased human recipient was negative for PERV A, B, and C post-transplant suggesting that NO disease transmission occurred. In addition, a study of pig islets into humans out of New Zealand has demonstrated no PERV transmission with 7 years of post-transplant follow-up.

BG: This remains an uncertain but theoretically real risk. Our animals are not PERV free. We have an opt out ability for intimate contacts (very few did so), a major surveillance program, and contact precaution in force.

RM: There has never been a transmission of PERV to a human despite more than 200 patients having received living porcine cells and tissue. Close surveillance has become an acceptable framework for zoonotic management.

BR: When the field was starting, PERVs were considered as a major issue. It has only been possible to infect human cells *in vitro* with PERV, and under very specific conditions. In the few human trials of xenogeneic islet transplantation, no PERV transmission could ever be documented. Interestingly, anti-PERV antibodies could not be found in a serological study of slaughterhouse butchers, who literally “bathe” in porcine blood! (3) PERV A and B exist in low copies and are not considered as dangerous to the humans, so only PERV C should be controlled. I believe that eGenesis (note: a US company developing gene-edited pigs for xenotransplantation) has the technology to target PERV genes but does not consider it necessary to knock off these genes because it may lead to off-target effects.

**Figure:**
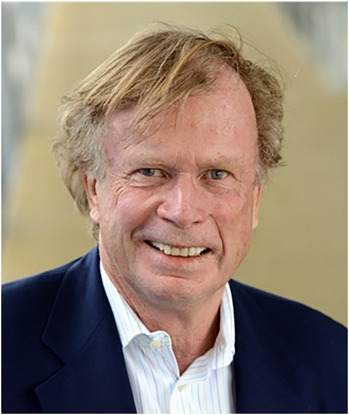
Dr. Bruno Reichart (BR).

One infectious consideration regards transmission of porcine CMV. This virus from the herpes family is very different from human CMV and no drug is effective against it. Porcine CMV transmission has been associated with high lethality in pig-to-primate experiments. Fortunately, it is very easy to breed and maintain porcine CMV-free animals.

**Q: Dr. Locke, Dr. Montgomery: While both of you have reported urinary output, in the Alabama case this was not associated with creatinine clearance. Was it the same in the NY cases? What would be an explanation? Was it just a matter of timing? Could you prolong the experiment beyond 3 days (from regulatory and ethical standpoints)?**

JL: Kidney function was not a primary outcome for our study at UAB. Our recipient had already been brain dead for 5 days prior to enrollment in our study and was undergoing the pathophysiologic derangements associated with brain death. It is unlikely that extending the time frame in a preclinical human model of brain death will yield more information on kidney function as brain death physiology worsens with time. It is also important to place kidney function in this model in the context of what is known in human-to-human allotransplantation. Specifically, human recipients of kidneys from human brain-dead donors often experience delayed graft function after transplant, which is characterized by no to minimal urine output or renal clearance necessitating dialysis in the first post-transplant week. Thus, lack of urine output and renal clearance are common early after human-to-human deceased donor kidney transplantation.

RM: After implanting our two kidney xenografts we saw more than a doubling of eGFR but we did not remove the native kidneys.

**Q: Dr. Montgomery, Dr. Locke: what boxes still have to be ticked before you move to actual kidney xenotransplantation? Are you preparing to perform a first case?**

JL: At UAB, we are in conversation with the FDA regarding an IND (investigational new drug) approval for the 10 GE pig kidney. Once we have an IND, it will approve us for a phase I clinical trial. We have 10 GE pigs at our pathogen free facility that will be of size between March and June of this year. Our goal is to start our phase I clinical trial during that time frame.

RM: I think through the Maryland heart transplant eIND (emergency IND) process we were able to get a clearer picture of what the FDA is looking for in terms of milestones for them to grant permission for phase I trials. We are working towards these goals.

**Q: Dr. Griffith: what follow-up will you require on your first patient before you decide the time has come for a second case?**

BG: We are approved for 1 case by the FDA. Revivicor/United Therapeutics plans a formal multi-institutional IND as soon as possible, but expect it may take up to 2 years for FDA approval. We have already had an INTERACT (note: informal non-binding consultation with the Center for Biologics Evaluation and Research at the FDA) pre-IND meeting. I believe several months should pass before we know whether an additional few cases might be indicated. That said, we have already learned a great deal about xenoheart care. Surely, should additional expanded access cases be deemed reasonable, they will add to the knowledge necessary for the best formal IND study.

**Q: How would you judge the risk of late rejections, antibody-mediated rejection (AMR) and chronic inflammation in xenotransplantation of the heart and the kidney?**

BG: I am comfortable we can deal with AMR but must prevent it rather than have to treat it. The pigs are edited for reduced inflammatory response. Unlike our animals we will be using endomyocardial biopsies, cell-free DNA testing, and allo-mapping, frequent stress echocardiography, and advanced immunosuppressant drug monitoring. We will learn.

JL: These are great questions that will be difficult to answer without moving into living persons. This should be done in the context of a clinical trial.

RM: In addition to the α 1-3 Gal KO, we also used a “thymokidney,” of which the Columbia University team has published promising results demonstrating tolerogenic effects of autologous pig thymic transplantation under the capsule of the xenograft. We believe that this innovation will help further protect the pig kidney from late rejections and chronic AMR.

**Q: Dr. Reichart: How would you comment on the fact that a clinical xenogeneic heart transplant could be performed, while for the kidney decedent recipient models still had to be used?**

BR: I strongly believe that the heart is an easier organ than the kidney for xenotransplantation. There have been consistent observations of prolonged heart transplant survival in pig-to-primate models, including in Munich. This has not been the case for the kidney, although I am admirative of Dr. J. Markmann’s recent reports with kidney xenotransplants (4). The reason is probably that the kidney is a much more complex organ than the heart from a purely functional standpoint. David Cooper would disagree with me and is a strong advocate for going to kidney first, notably because of the much lower technical complexity of the procedure (no heart-lung machine necessary). At the end of the day, the reality is that the heart was first!

**Q: Dr. Reichart: What made it possible to do this first clinical xenoheart transplant in the United States?**

BR: I think the major reason is that the institutional commitment was extremely strong. I think that there was a strong will to be first to do it. This is one reason why they opted for a cloned rather than farmed animal. This has produced a single 10 GE animal, but much faster. This strategy can be applied for a small number of initial cases but is not sustainable. There is a very emotional mentality in the United States, that is not really seen in Europe. Obviously, the money was there, but financial considerations were not the major issue, in contrast to the perspective of a groundbreaking achievement. From a regulatory standpoint, the FDA has been very responsive. The transplant was done on a compassionate protocol, which made it possible without the very stringent rules applied by the FDA for clinical trials.

It is very encouraging to see that worldwide, the reporting by the lay press has been overall very favorable and that the public has received the news with enthusiasm and admiration. There has been very little opposition in the society. In Germany, journalists form major newspapers wrote articles in support of the ethical acceptability of the procedure.

**Q: Dr. Reichart: What is your appraisal of the xenokidney experiments and their results?**

BR: I wonder how relevant these experiments have been for advancing xenotransplantation of kidneys. In my opinion, it is necessary to first demonstrate consistently predictable long-term success in preclinical studies in non-human primate models before moving to the clinic. The major achievement of these experiments is that a line was crossed with unquestionable acceptance by the society. The ethics of utilizing organs from animal origin should not be underestimated, and earlier attempts at utilizing baboon hearts were not accepted at the time. The population widely accepts to use of pigs, which are obviously an important source of food, as xenogeneic organ donors.

**Q: What will happen next?**

RM: More focused primate work with the exact pig construct and immunosuppression that the group wants to move forward to phase, I trials and more studies on the recently deceased.

BR: The Maryland case is a great landmark in the history of transplantation, and I have personally congratulated Dr. Griffith for his, and his team’s achievement. The move to subsequent cases is potentially tricky. Several heart transplant programs will want to perform porcine heart transplantation, but this will have to be done with extreme caution. In the year following the Barnard’s first heart transplant in 1967, many programs opened throughout the world, with dismal survival results. This led to a near-stopping of this activity that lasted for about a decade.

Regarding xenotransplantation in general, upscaling the procedure will require to move from cloned animals to dedicated breeding farms. This will be extremely costly, since the breeding of these genetically modified pigs will require pathogen-free facilities with high standards defined by regulatory agencies: microbiological filters, showers, masks and clean room gowning, autoclaved food,… The number of facilities will be naturally limited. I envision that there will be no more than 1–2 facilities per continental region (North America, Europe,…), working in close interaction with their respective regulatory agencies (FDA, EMA,…).
